# Notable fibrolytic enzyme production by *Aspergillus* spp. isolates from the gastrointestinal tract of beef cattle fed in lignified pastures

**DOI:** 10.1371/journal.pone.0183628

**Published:** 2017-08-29

**Authors:** Flávia Oliveira Abrão, Eduardo Robson Duarte, Moisés Sena Pessoa, Vera Lúcia dos Santos, Luiz Fernando de Freitas Júnior, Katharina de Oliveira Barros, Alice Ferreira da Silva Hughes, Thiago Dias Silva, Norberto Mário Rodriguez

**Affiliations:** 1 Instituto Federal Goiano, Ceres, Brasil; 2 Instituto de Ciências Agrárias da Universidade Federal de Minas Gerais (UFMG), Montes Claros, Brasil; 3 Universidade Federal de Goiás, Goiânia, Brasil; 4 Instituto de Ciências Biológicas da UFMG, Belo Horizonte, Brasil; 5 Escola de Veterinária da UFMG, Belo Horizonte, Brasil; Hubei University, CHINA

## Abstract

Fungi have the ability to degrade vegetal cell wall carbohydrates, and their presence in the digestive tract of ruminants can minimize the effects of lignified forage on ruminal fermentation. Here, we evaluated enzyme production by *Aspergillus* spp. isolates from the digestive tracts of cattle grazed in tropical pastures during the dry season. Filamentous fungi were isolated from rumen and feces by culture in cellulose-based medium. Ninety fungal strains were isolated and identified by rDNA sequence analysis, microculture, or both. *Aspergillus terreus* was the most frequently isolated species, followed by *Aspergillus fumigatus*. The isolates were characterized with respect to their cellulolytic, xylanolytic, and lignolytic activity through qualitative evaluation in culture medium containing a specific corresponding carbon source. Carboxymethyl cellulase (CMCase) activity was quantified by the reducing sugar method. In the avicel and xilan degradation test, the enzyme activity (EA) at 48 h was significantly higher other periods (*P* < 0.05). Intra- and inter-specific differences in EA were verified, and high levels of phenoloxidases, which are crucial for lignin degradation, were observed in 28.9% of the isolates. *Aspergillus terreus* showed significantly higher EA for avicelase (3.96 ±1.77) and xylanase (3.13 ±.091) than the other *Aspergillus* species at 48 h of incubation. Isolates AT13 and AF69 showed the highest CMCase specific activity (54.84 and 33.03 U mg^-1^ protein, respectively). Selected *Aspergillus* spp. isolates produced remarkable levels of enzymes involved in vegetal cell wall degradation, suggesting their potential as antimicrobial additives or probiotics in ruminant diets.

## Introduction

Fibrous vegetation is the basis of the ruminant diet, and degradation of the cell wall carbohydrates in plant fibers is fundamental to ruminant digestion [1]. The symbiotic interactions between rumen microorganisms and ruminants can supply proteins, energy, and vitamins to the animal host, which contribute to their growth and production [2].

The plant cell wall is composed of cellulose (35–50%), hemicellulose (30–35%), and the phenylpropanoid polymer lignin (10–25%) [3]. In semiarid regions, the quantity and quality of available pasture are compromised during dry periods when forage digestibility is reduced by the physiological lignification of the vegetative cell wall [4].

Fungi produce lignocellulolytic enzymes and contribute synergistically to the decay of lignocellulosic residues in nature. If the lignin complex is removed, cellulose and other carbohydrates can be released for use in industrial processes and to meet the nutritional needs of ruminants [5]. Fungi also produce enzymes that facilitate polysaccharide degradation in ruminants, and cultures of *Aspergillus oryzae* and their extracts have been used as supplements in ruminant diets to improve productivity [6].

Anaerobic fungi in the rumen may be important for tropical forage degradation, as they produce enzymes that hydrolyze the cellulose and hemicellulose in lignified pastures [7, 8]. However, little is known about the population of aerobic fungi that naturally occur in the bovine rumen.

An earlier study of the cellulolytic activity of fungi in the digestive tract of dairy cattle revealed *Aspergillus* and *Paecilomyces* isolates that degrade microcrystalline cellulose [9]. In addition, rumen fluid from beef cattle fed only in lignified tropical pastures predominantly contained isolates of the genus *Aspergillus* [10], which have shown potential for supplementation of animal diets and industrial purposes [11, 12]. Supplementation of ruminant diets with microbial additives containing exogenous fungi and their enzymes has been reported to increase milk production by optimizing digestion [13, 14].

We believe that, the rumen of cattle fed exclusively in lignified pastures can be a source of fungi strains showing high-level production of enzymes that degrade lignified cell wall. The inclusion of selected autochthonous fungi or their hydrolytic enzymes during dry periods could favor the degradation of plant cell walls of forages, thus reducing production costs. In the present study, we evaluated the enzyme activity (EA) of *Aspergillus* spp. isolates from the digestive tract of cattle grazing on lignified tropical pasture to select strains with the optimal ability to degrade plant cell walls.

## Materials and methods

### Strain collection

Samples were collected from Nellore (Zebu) beef cattle on farms in Montes Claros and Coração de Jesus in northern Minas Gerais State, Brazil. This region is located at approximately 16° 51' S and 44° 55' W, and it has a humid tropical climate with a dry summer (As), according to the Köppen classification [15], marked by a dry season from May to September and a rainy season from December to February.

The study evaluated 113 cattle: 32 adult cows, 31 steers (24–40 months), and 50 calves (6–8 months). The cattle were raised in an extensive system on *Brachiaria* spp. pasture, supplemented with a mineral mixture for beef cattle, containing urea, according to age category.

Rumen fluid and feces were collected during the dry season, which had an average rainfall of 136.9 mm according to the Fifth District of Instituto Nacional de Meteorologia of Brazil. After eight hours of fasting, calves were immobilized in a restraint chute to obtain rumen fluid using a sterile catheter as described by Abrão et al. [10]. After eight hours of fasting, steers and cows were slaughtered by brain concussion and bleed in a slaughterhouse under sanitary inspection. Approximately 15 mL of rumen fluid was collected through an incision in the ventral rumen sac. Fecal samples were obtained directly from the rectal ampulla with a sterile swab. All samples were transported to the laboratory at 4°C and stored for up to 1 h in sealed sterile test tubes [10].

All procedures in this study were approved by the Ethics Committee on Animal Experimentation (CETEA) of the Federal University of Minas Gerais (UFMG; registration no. CETEA 128/2013), which is regulated by the National Council for the Control of Animal Experimentation of Brazil.

### Isolation and identification of fungi

Rumen fluid and feces were inoculated with sterile swabs onto C medium agar plates containing 1% microcrystalline cellulose (Avicel), 0.5% ammonium sulfate, magnesium sulfate heptahydrate 0.05%, and 2% agar-agar in distilled water [16].

The fungal genera of the **110** mycelial isolates were determined by microculture tests. Micro-morphological characteristics were observed under an optical microscope and were compared to those described for fungi of biotechnological and veterinary interest [17]. Positivity rates were compared by the χ^2^ test using the R statistical program (v.3.2.2).

After the fungal cultures grew, they were replated on C agar medium in isolate tubes for enzyme assays [18]. These isolates were preserved by the Castellani method and were stored at room temperature [19].

### Molecular identification

One hundred and ten isolates were identified by molecular biological methods. *Aspergillus* spp. with black pigmentation (N = 13) were identified by microculture test [17] due to difficulties in DNA extraction and PCR amplification.

Filamentous fungi were grown on Sabouraud agar for seven days, and DNA was extracted according to the method of Rosa et al. [20]. The ITS region of rDNA was amplified from the extracted DNA by polymerase chain reaction (PCR) using primers ITS1 (TCCGTAGGTGAACCTGCGG) and ITS4 (TCCTCCGCTTATTGATATGC), according to the method of White et al. [21]. The amplified product was quantified with a NanoDrop 1000ND (NanoDrop Technologies), and the concentration was adjusted to 100 ng μL^-1^ for use in sequencing reactions.

Sequencing was performed with DYEnamic (Amersham Biosciences, USA) in a MegaBACE 1000 automated sequencing system at the Genome Analysis Center and Gene Expression of UFMG. The obtained DNA sequences were analyzed using BLASTn (v.2.215) of BLAST 2.0 at the NCBI website [22]. Isolates with ≥99% sequence similarity to deposited sequences were considered the same species.

### Qualitative enzyme assays on solid media

#### Cellulolytic and xylanolytic activity assay

After incubation at 37°C for seven days in C medium agar, fungus colony discs (5-mm diameter of each fungi isolate were seeded, in triplicate, at the center of Petri dishes (140 x15 mm) containing **30 mL** of C agar medium containing 1% microcrystalline cellulose (Avicel) as the sole carbon source as previously described. To assess xylanolytic activity, fungal inoculates were seeded onto medium containing 1% xylan, 0.5% ammonium sulfate, 0.05% magnesium sulfate heptahydrate, and 2% agar-agar in distilled water.

The three replicates were incubated at 37°C, and EA activities were evaluated by a method adapted from Theater and Wood [16]. After incubation, plates were washed with 30 mL of Congo red solution (1 mg mL^-1^) for 15 min. Excess dye was removed by rinsing three times with 15 mL of 1 M NaCl solution. After 2 h, the diameter of the clearing around the colony, indicative of cellulose or xylan degradation, was measured. The enzyme activity (EA) index at 24, 48, 72 h and 96 h were calculated by dividing the diameter of the hydrolysis zone by the colony diameter [23]. The diameters of each plate were measured at eight equidistant points to produce a mean.

The Wilcoxon nonparametric test, at 5% significance, was used to compare mycelial fungal EA indices according to carbon source. Concomitantly, the EAs of each species and incubation period were analyzed using the non-parametric Kruskal–Wallis test, at 5% significance. We evaluated the correlation between the EA indices for xylan and cellulose by Spearman correlation. The R software package (v.3.2.2) was used for data analysis.

#### Ligninolytic activity

Fungal isolates were inoculated in triplicate on agar medium containing 15 g of malt extract, 1 g of dextrose-peptone, 5 g of gallic acid, and 20 g of agar in 1 L of distilled water as previously described [24].

After incubation at 37°C for 5 days, hydrolysis, as indicated by color intensity, was classified as weak, moderate, or intense, according to Conceição et al. [25]. For negative controls, plates containing the medium without a fungal isolate were incubated. The χ^2^ test was used to evaluate the results using R (v.3.2.2).

#### Quantitative carboxymethyl cellulase (CMCase) assay in liquid medium

The 20 *Aspergillus* spp. isolates showing the highest EA indices in the previous step were selected for quantification of CMCase activity in a completely randomized test with three replicates.

The selected fungal isolates were grown in yeast nitrogen base medium (Sigma-Aldrich) containing 1% sodium carboxymethyl cellulose in sodium acetate buffer (50 mM, pH 5.5) at 30°C for 48 h. Then, the culture was centrifuged at 5,000 × *g* for 15 min, and the supernatant was used to quantify EA based on reducing sugar measurement, according to the 3,5-dinitrosalicylic acid (DNS) method as described by Miller [26].

The enzyme assays were performed in 96-well plates. The reaction mixture, containing 40 μL of 50 mM sodium acetate buffer (pH 5.0), 10 μL of microbial culture supernatant, and 50 μL of 1% sodium carboxymethyl cellulose, was incubated in a thermal cycler (Veriti 96-Well; Applied Biosystems) at 50°C for 30 min. The mixture was then cooled to 4°C to stop the reaction, and 100 μL of DNS solution (1% DNS, 1% sodium hydroxide, and 20% potassium sodium tartrate) was added. The microplates were incubated at 99°C for 5 min and at 4°C for 15 min. Then, the absorbance at 550 nm was determined on a microplate reader (Thermo Scientific MultiSkan Spectrum). For the control, DNS solution was added only at the 0 time point [26].

All samples were tested in triplicate, and a standard glucose curve was constructed using 0–270 μM glucose solutions. One unit of cellulase activity was defined as the quantity required to produce 1 μmol of glucose per microgram of total protein per minute (1 U = μmol min^-1^), as previously described [26, 27]. EA was expressed as U mL^-1^.

Fungal biomass was based on the dry weight obtained after filtration through Whatman No. 2 filter paper followed by drying to a constant weight [28]. The protein concentration of the supernatant was determined by the Bradford method using bovine serum albumin as a standard. The standard curve was fit to the absorbance at 595 nm of protein concentrations of 0, 25, 50, 100, 200, and 300 μg mL^-1^ measured in a microplate reader [29]. After exploratory data analysis, the variables were compared using the nonparametric Kruskal–Wallis test at 5% significance level in R (v.3.2.2).

## Results

### Positivity and identification of cellulolytic filamentous fungi

**Fig 1:**
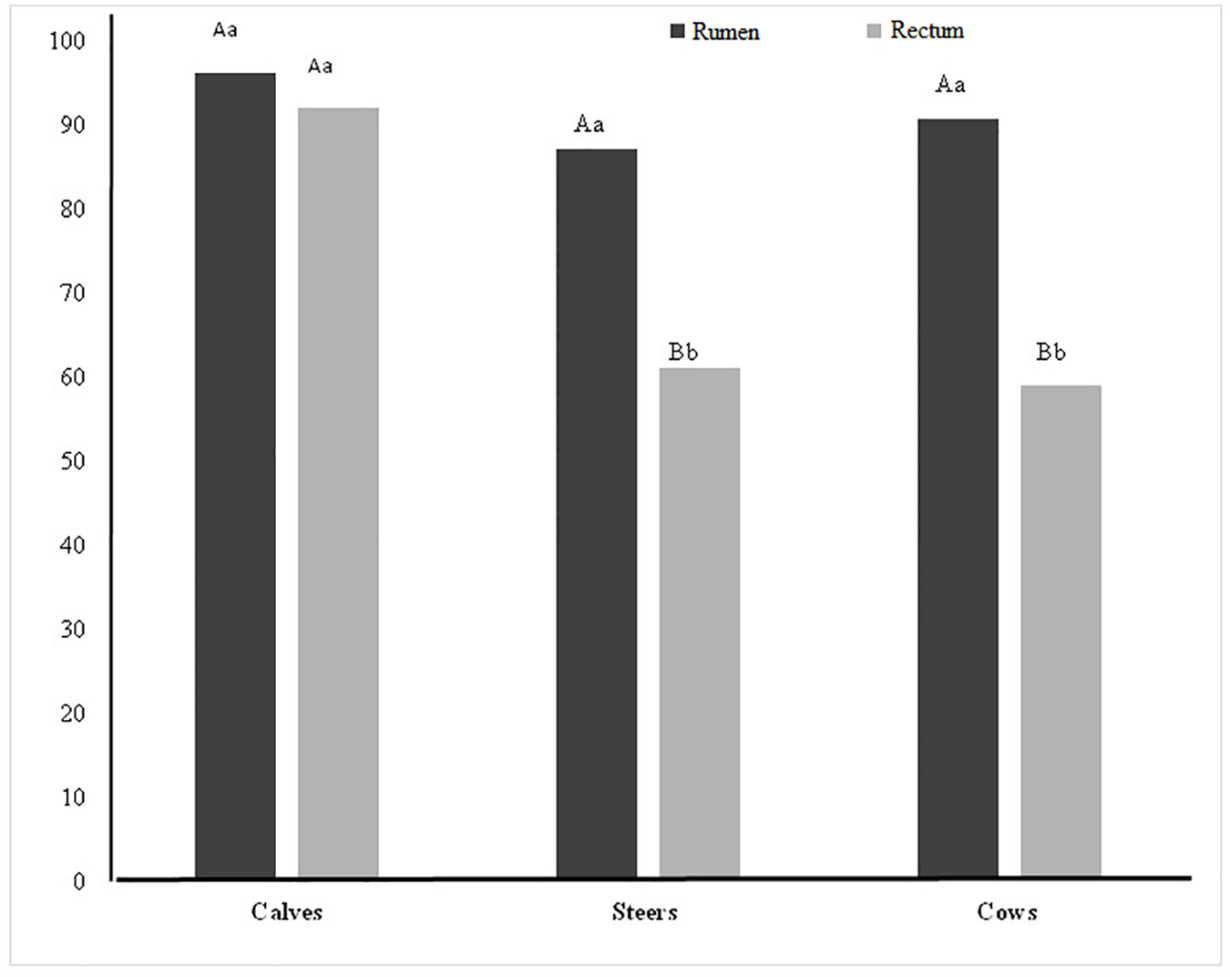
Detection rate of cellulolytic fungi (%) in samples obtained from the gastrointestinal tract of beef cattle that grazed on tropical lignified pastures. Lowercase letters indicate significant difference between organ origins and uppercase letters indicate significant difference between categories as determined by χ^2^ test with a 5% significance cutoff.

Aerobic filamentous fungi that can grow on a medium containing cellulose as the sole carbon source were detected in 60–96% of the cattle, and ruminal detection rate was not influenced by animal age. However, compared to rectum samples this rate was higher in calves than in cows and steers. The positivity was higher in ruminal samples than rectal samples, when considering the adult cattle (Fig 1, *P* < 0.05).

**Fig 2:**
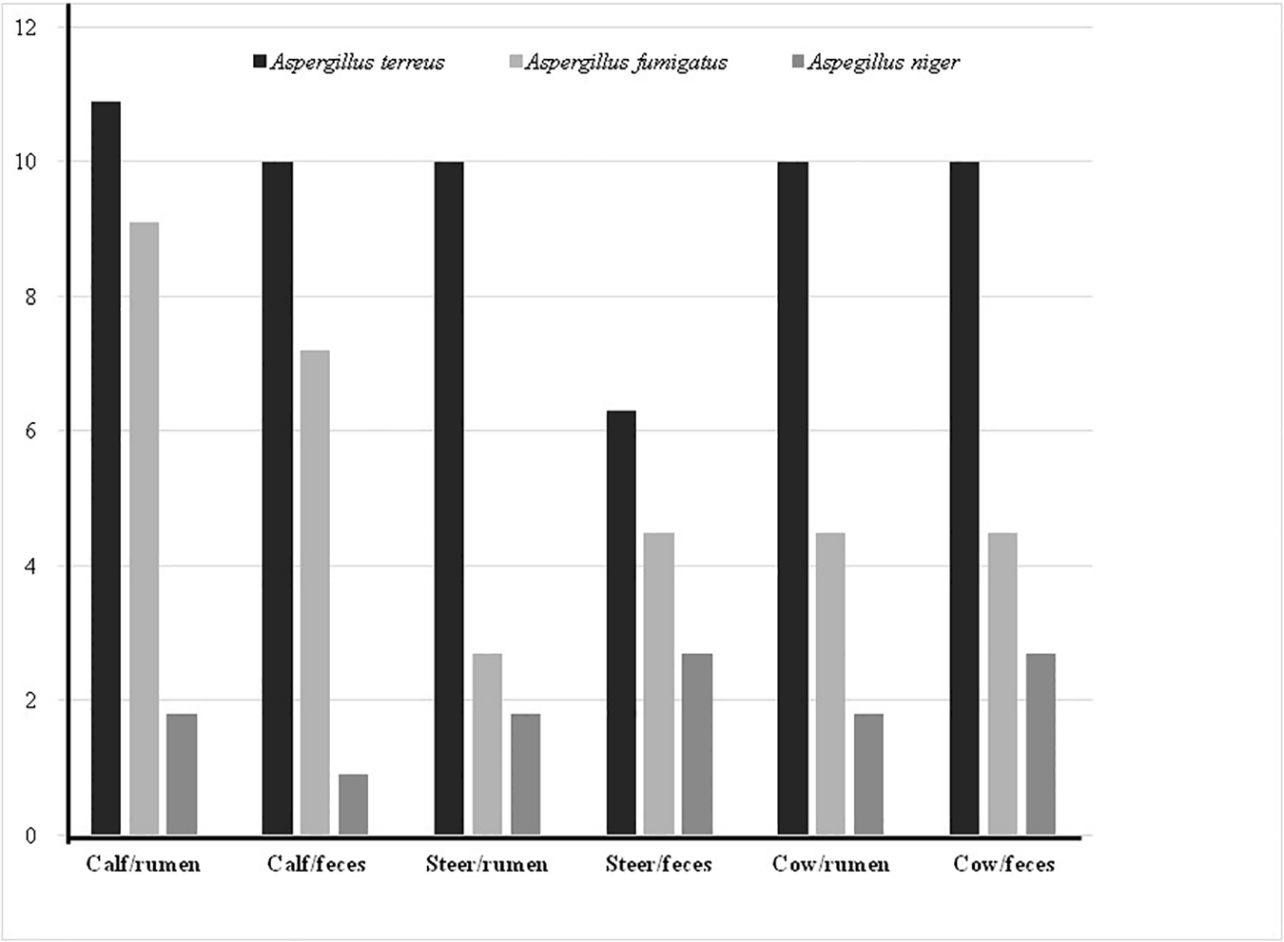
Distribution of *Aspergillus* species (%) from the rumen and rectal ampulla of cattle that grazed on *Brachiaria* sp. during the dry season.

Sequencing of rRNA gene fragments revealed 34 isolates of *Aspergillus fumigatus* (100% identity, GenBank accession number: KF781534) and 63 isolates of *Aspergillus terreus* (100% identity, GenBank accession number: KF781532). Thirteen black fungal isolates were not identifiable by molecular techniques because of low quality DNA after repeated extraction and amplification attempts. For these isolates, identifications were made by macro-morphological characteristics observed on potato agar medium and micro-morphology revealed by microculture. The characteristics of this fungus clearly corresponded with the characteristics of *Aspergillus niger* (Fig 2). Considering the total number of fungi isolated, the most frequently occurring species was *A*. *terreus* (*P* < 0.05), followed by *A*. *fumigatus* and A. *niger* for the tree bovine category.

### Qualitative assay of enzyme production on solid medium

**Fig 3:**
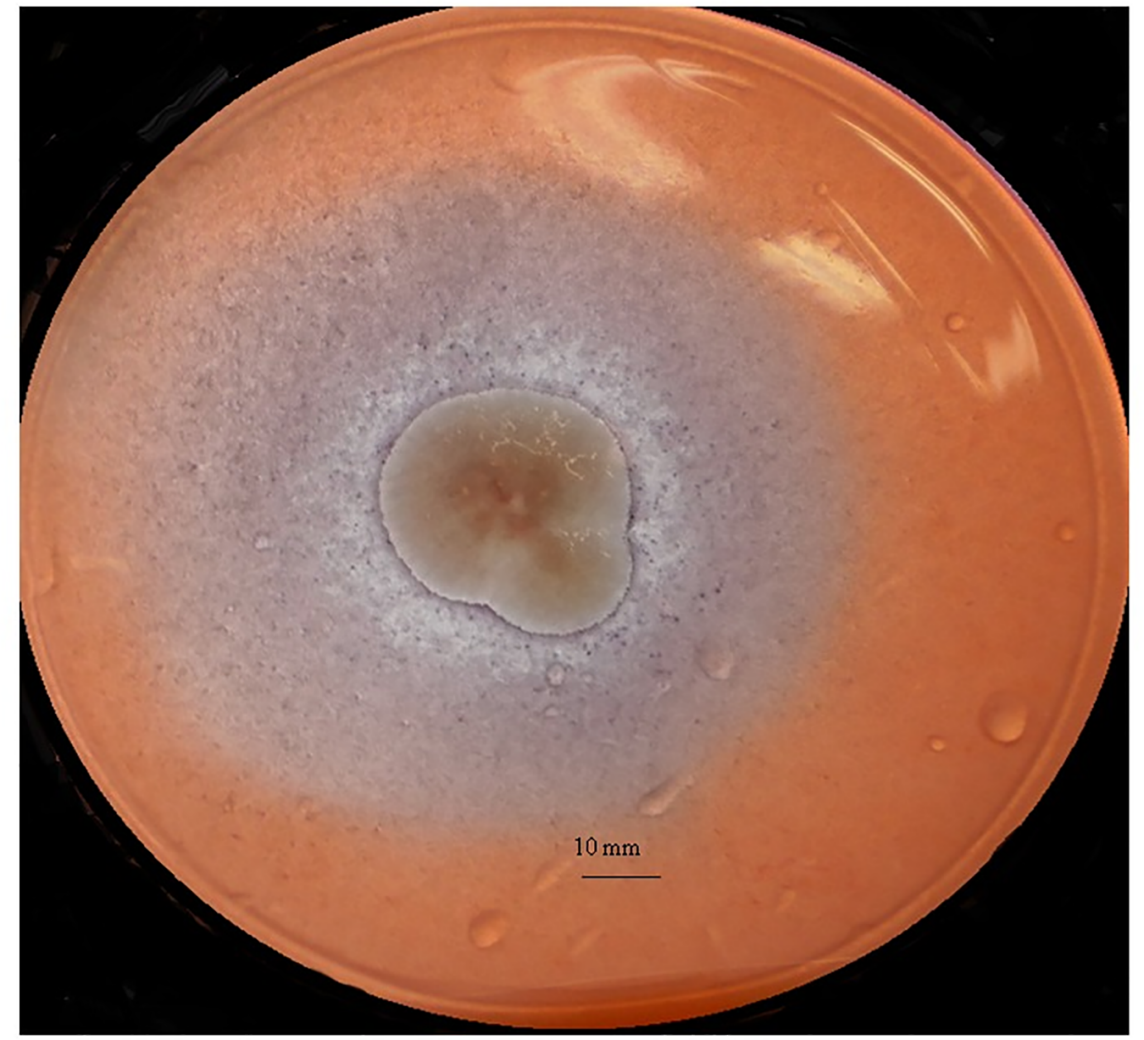
Avicel degradation at 48 h by *Aspergillus terreus* isolated from cow rumen fluid.

In this study, the mean EA of avicelase and xylanase at 48 h was higher (*P* < 0.05) than at other time periods and significantly higher than the xylanase EA at 48 24 h (*P*<0.05), which indicates no correlation between the activity of theses enzymes (Table 1). *Aspergillus terreus* showed significantly higher EA for avicelase (3.96 ±1.77, Fig 3) and xylanase (3.13 ±.091) than the other *Aspergillus* species after 48 h of incubation.

**Table 1:** Mean enzyme activities (EAs) of *Aspergillus* spp. isolated from the gastrointestinal tract of beef cattle that grazed on tropical pastures.

Enzymes	EA 24 h	EA 48 h	EA 72 h	EA 96 h
Avicelase	2.51±1.22^Aab^	3.51±2.01^Ab^	2.00±0.32^Aa^	2.03±0.43^Aa^
Xylanase	2.66±1.40^Aab^	2.80±1.00^Bb^	2.04±0.15^Aa^	2.04±0.28^Aa^

Note: Lowercase superscripts in row indicate significant difference by Kruskal-Wallis test and uppercase superscript in column indicate significant difference by nonparametric Wilcoxon test (*P*<0.05). EA = hydrolysis zone diameter/colony diameter.

**Fig 4:**
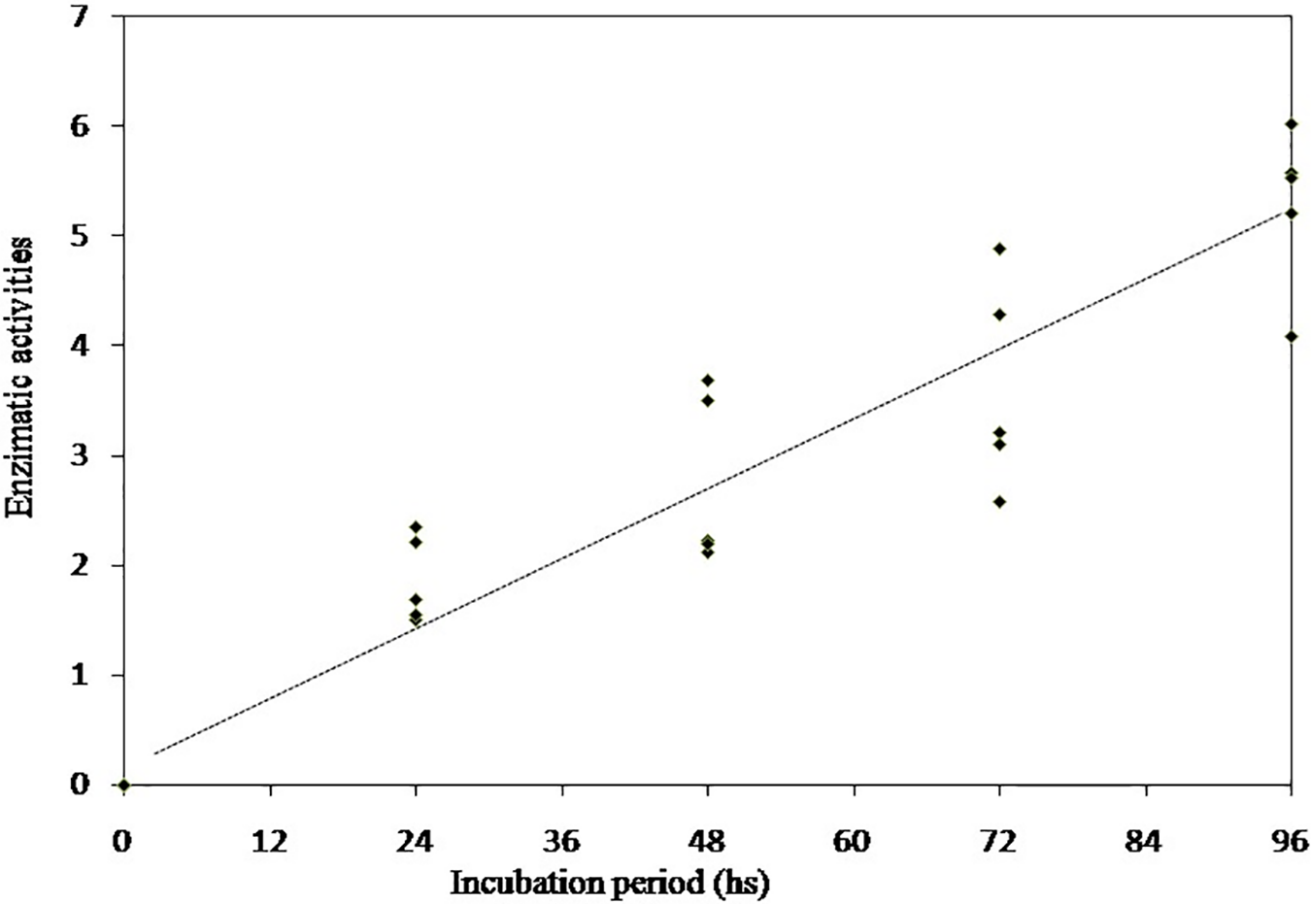
Linear increase of the avicelase activity for five selected *Aspergillus terreus* isolates from rumen fluid collected from Nellore cows. Avicelase activity = hydrolysis zone diameter/colony diameter.

In order to demonstrate intraspecific differences for avicelase activity, five isolates of *A*. *terreus* from cow rumens showed higher EA indexes than other evaluated fungi (Fig 4, *P<*0.05). The EA showed linear increase during the evaluated periods (R = 0.92).

A large number of fungal isolates produced phenoloxidases (*P* < 0.05). In fact, only 8.9% of the isolates did not produce phenoloxidases, whereas 38.9%, 23.3%, and 28.9% showed weak, moderate, and intense production, respectively.

### Quantification of CMCase activity

The concentration of CMCase produced varied significantly among the species and strains tested (Table 2), demonstrating both inter- and intra specific differences. The EAs of isolates AT13 and AF69 was high, and the enzymes in these isolates were capable of converting substrate to product faster and more efficiently than the enzymes in other isolates.

**Table 2:** Quantification of carboxymethyl cellulase (CMCase) activity produced by *Aspergillus* spp. isolates from the gastrointestinal tract of cattle that grazed on lignified pastures.

Isolates	Concentration (U ml^-1^ extract)	Specific activity (U mg^-1^ protein)	Productivity (U mg^-1^ biomass)
AT7	27.73± 7.64^ab^	0.69±0.25^ab^	1456.93±344.58 ^ab^
AT8	18.87± 0.58^ab^	0.45± 0.03^ab^	770.33±274.71 ^ab^
AT13	22.34±4.03^ab^	54.84±10.99^a^	1094.81±347.68 ^ab^
AT15	43.26±15.06^a^	1.08±0.38^ab^	2216.34±502.33 ^a^
AT17	7.68±1.03^ab^	18.44 ±3.01^ab^	369.78±65.91 ^ab^
AT19	23.51±4.67 ^ab^	0.65±0.23 ^ab^	852.16 ±175.59^ab^
AT22	35.62±16.48 ^ab^	0.61±0.23^ab^	224.41±206.88 ^ab^
AT40	7.99±0.35 ^ab^	20.03±1.28 ^ab^	374.24±42.64 ^ab^
AT42	39.43±20.90 ^ab^	0.66±0.41^ab^	1662.86±881.04 ^ab^
AT43	1.26±0.05 ^b^	0.02±0.002^b^	58.25±1.65 ^ab^
AT45	12.00±10.50 ^ab^	0.26± 0.24^ab^	503.57±455.1 ^ab^ 4
AT46	28.06±9.69 ^ab^	0.66±0.33^ab^	81.74±28.68 ^ab^
AT50	20.09±2.04 ^ab^	0.46±0.05^ab^	830.97±83.69 ^ab^
AT51	29.11±2.99 ^ab^	0.89±0.16^ab^	1275.86±229.25 ^ab^
AT52	9.43±1.12 ^ab^	23.08 ±3.24^ab^	232.54±375.06 ^ab^
AT53	6.16±0.46 ^ab^	0.17±0.025^ab^	17.52 ±1.18 ^b^
AN54	21.57±1.32 ^ab^	0.52±0.022^ab^	893.12±64.04 ^ab^
AF69	13.66±1.35 ^ab^	33.03±4.43 ^a^	271.95±21.08 ^ab^
AT79	24.23±1.82 ^ab^	0.42± 0.06^ab^	1059.78±219.91 ^ab^
AF83	20.99±20.29 ^ab^	0.41±0.55^ab^	63.20±90.02 ^ab^

Note: Different superscript letters in a column indicate significant difference by the nonparametric Kruskal–Wallis test at 5% probability. AT = *Aspergillus terreus*; AN = *Aspergillus niger*; AF = *Aspergillus fumigatus*. U = quantity of enzyme required to produce 1 μmol of glucose per microgram per minute (U = μmol ml^-1^ min^-1^).

## Discussion

### Identification and quantification of cellulolytic filamentous fungi

The aerobic filamentous fungal isolates from Nellore cattle fed on lignified pastures grew well in cellulose-containing medium, and their detection rate was greater than 90% for rumen samples and not influenced by age. This shows the relevance of this microorganism. The detection rate was lower in the rectum of cows and steers, and we suggest that a more stable microbiota of the large intestine of adult cattle resulted in a reduction in these fungi.

In another study, the positive rate in the C medium for dairy cows fed on pasture during the rainy season was significantly lower (86.7%) when compared to the rates observed for cows fed with sorghum silage (100%). The tender pasture in the rainy season had lower fiber contents than the silage, explaining this lower rate [18].

In this study, *Aspergillus* spp. was the only genus isolated, and *A*. *terreus* was the most frequently identified species. A similar study using Sabouraud agar medium also demonstrated the predominance of *Aspergillus* spp., which represented 56% of the rumen fungal isolates from dairy cows fed sorghum silage or on *Brachiaria brizantha* pasture [18]. In our previous study of Nellore beef cattle that fed only on lignified tropical pastures, a Sabouraud agar test showed higher frequencies of aerobic fungi in the rumen of cows and calves than in the rumen of steers, and a significantly higher frequency in cows than in calves. *Aspergillus* spp. was also the most frequent genus among the isolates from rumen fluid. *Aspergillus fumigatus* and *A*. *terreus* were observed in all bovine categories [10].

In the analysis of fungal population in the rumen contents of steers raised on lignified pastures or in feedlots without forage and after microculture, the genus *Aspergillus* was the most frequently identified, and *A*. *terreus* was detected in both bovine groups [30].

In a study of the aerobic fungi of the ruminal fluid of five cows, five sheep, and five goats from Nigeria, a higher proportion was detected in the samples from cows than other ruminants. The genera *Mucor*, *Aspergillus*, *Fusarium*, and *Penicillium* were detected. However, *Mucor* spp. (40.6%) was the most common genus among the fungi isolates [31]

*Aspergillus* isolates can be selected by their versatility and efficiency in catabolizing different carbon sources [32]. All fungal isolates in this study showed cellulolytic activity and have potential for use in biotechnological applications. Reports on the effects supplementation with microbial enzymes on animal productivity have been inconsistent, possibly due to the complexity of factors affecting enzyme activity and enzyme-substrate interactions [33].

### Qualitative analysis of cellulose xylanase and lignin hydrolytic enzyme production on solid medium

Avicelases play a fundamental role in the bioconversion of agricultural wastes to useful products [34]. Theses enzymes can catalyze most of the bond-cleavages in the saccharification of crystalline cellulose and is one of the major components of cellulase preparations, especially for fungus derived commercial enzymes [34]. Of the number of microorganisms producing cellulases, the report of avicelase production is remarkably scarce in comparison to that of endoglucanase and beta glucosidase [35].

In this study, avicelase EA was higher at 48 h than at 24 h of incubation, probably due to an adjustment period (or lag phase) in fungal growth on cellulose. This adaptation can be explained by the production fungal cellulase only occurring in the presence of its substrate [36].

For the evaluated fungal strains, *in vitro* avicelase production did not depend on xylanase production, and 28.9% of these fungal isolates showed high production of phenoloxidases, which solubilize lignin. High phenoloxidase production could promote the degradation of cell wall carbohydrates in lignified pasture vegetation during the dry season.

Many *Aspergillus* species are known to be good producers of cellulases [37], but few research reports are available on the production of cellulase from *Aspergillus terreus* [38].

The EA of avicel degradation observed in this study for the five selected *A*. *terreus* isolates from cows was higher than that reported for other aerobic fungi. For aerobic mycelial fungi isolated from dairy cattle fed no lignified tropical forage, the genus *Aspergillus* showed higher avicelase EA compared to the *Rhizopus* genus. Eight isolates of *Aspergillus* spp. and six of *Paecilomyces* spp. showed EA ≥1.0, indicating potential for utilization in ruminant nutrition. However, only one *Aspergillus* isolate showed EA greater than 2.0 after 48 h of incubation [9]. In a study of eight mycelial fungi strains isolated from soils in the Brazil, the highest value for EA was 1.847 after 96 h of incubation [39].

*Aspergillus* spp. are well known for production of extracellular cellulases and has been the predominant genera in agricultural wastes [35]. A study of twenty-nine fungal strains that were isolated from agricultural wastes evaluated the cellulase production on different waste substrates. The isolated strain *Aspergillu*s MAM-F23 gave the highest avicelase activity (45 U/ml) on wheat straw [40].

In this study, the isolates of *A*. *fumigatus* from cattle rumen showed an avicelase EA of 3.2 at 48 h of incubation on a poor culture medium. Previously, an isolate of this species also produced avicelase from the solid-state fermentation of wheat straw at 55°C and pH 5.5 after 72 h [37].

Another *Aspergillus* species has also produced CMCase and xylanases. The EA of *Aspergillus caesiellus*, which is considered a moderate halophile, was evaluated using CMC as the sole carbon source. The highest production was observed on the sixth day of culture [41]. For *Aspergillus japonicus* isolates from soil, dried grass, and barnyard manure, the production of endocellulase and endoxylanase were stable from pH 4–7 and increased in the presence of copper and Magnesium [11].

In this study, intense phenoloxidase production was observed in 28.9% of the *Aspergillus* spp. isolates from bovine rumen, suggesting their potential for lignified forage degradation during the dry season. In another study, an isolate of *A*. *caesiellus* from sugarcane bagasse fermentation was not a lignin peroxidase producer [41]. The characterization of filamentous fungal isolates from soil producing lignin peroxidase reported showed that glucose inclusion favored the enzyme production [42].

### Quantification of CMCase activity

The enzymes in isolates AT13 and AF69 more efficiently converted substrate to product, and these isolates produced more units of enzyme per microgram of protein than the other isolates. Isolate AT15 incubated during 48 h showed the highest expression (43.26 U mL^-1^ of extract). Therefore, the potential of these fungal isolates for use as probiotics for cattle or additives to cattle feed should be assessed.

High CMCase expression may lead to efficient plant fiber degradation, and in this study, isolates of *A*. *terreus* and *A*. *fumigatus* from rumen fluid showed high CMCase activity. These findings differ from the results of previous studies, which reported the highest cellulolytic activity belonging to *Trichoderma* spp. and *A*. *niger* [43–45].

In other research, one selected isolate of *Aspergillus* sp. from agriculture wastes showed, after seven days of incubation, the highest CMCase production (233 U/mL) [40]. Gomes et al. [12] studied the kinetics of enzymes in *Aspergillus* spp. isolated for industrial purposes and reported 16.06–226.51 U of total cellulase activity/mg, highlighting the biotechnological potential of *Aspergillus* species.

However, in another study, *Aspergillus niger* had the lowest level of produced cellulase (0.300 U mL^-1^) when grown in culture medium containing sugarcane bagasse pretreated with NaOH [43].

The CMCase production observed in this study was higher than that reported during the fermentation of lignocellulosic wastes by *Trichoderma viride*, which produced 0.374 U mL^-1^ of cellulase and 0.776 U mL^-1^ of CMCase [46]. Khan et al. [47] observed an overall CMCase activity of 0.10 U mL^-1^ in *Trichoderma* spp. cultured with rice straw as a substrate.

One strain of *A*. *terreus*, M11, from Zhengzhou, China was also promising for cellulase production in short time periods. In solid-state fermentation of lignocellulosic materials, 581 U CMCase activity per gram of carbon source were obtained in solid-state fermentation. The cellulases were stable in acidic pH at 70°C and might be used as key enzymes in the production of bioethanol from cellulose [38].

Studies have demonstrated that supernatants derived from *A*. *terreus* cultures were free of mycotoxins, suggesting that it is safe for consumption [48]. Additionally, the sustainable potential of utilizing purified statins has led to a number of studies analyzing natural sources of statins for mitigating ruminant CH_4_ production [49].

Considering other possible benefits, lovastatin is produced by *A*. *terreus*, and it reduced total gas and CH_4_ production in a mixed culture of ruminant microorganisms *in vitro*. However, it did not alter H_2_ production. This metabolite decreased the total population of methanogens in ruminant culture, specifically lowering the numbers of Methanobacteriales and aerobic fungi [50]. These results point to the potential for future studies using *A*. *terreus* isolates from bovine rumen to mitigate CH_4_ production and improve the energy efficiency of ruminants fed in lignified pastures.

The high-level production of enzymes degrading lignified cell walls by these *Aspergillus* isolates of this study could reflect the selective pressure in the rumen of host cattle raised on tropical lignified pastures. The supplementation of diets with these fungi from rumen or their enzymes could promote more consistent production and adaptation to the conditions of the ruminal ecosystems of ruminants feeding on tropical lignified pastures.

## Conclusions

In this study, most *Aspergillus* spp. isolates from the bovine rumen were remarkable producers of cellulases, xylanases, and phenoloxidases for the degradation of lignin even after only two days of incubation. The activities of avicelase, xylanase, and CMCase showed evidence of inter- and intraspecific differences. Selected isolates of *A*. *terreus* and *A*. *fumigatus* from rumen fluid show potential use as probiotics for cattle fed in lignified pastures and for the commercial production of enzymes for biotechnology purposes.
